# Genomics in Epidemiology and Disease Surveillance: An Exploratory Analysis

**DOI:** 10.3390/life15121848

**Published:** 2025-12-01

**Authors:** Shraddha Tiwari, Thakur Dhakal, Baek-Jun Kim, Gab Sue Jang, Yeonsu Oh

**Affiliations:** 1 Department of Life Sciences, Yeungnam University, Gyeongsan 38541, Republic of Korea; shraddha_tiwari@yu.ac.kr (S.T.); thakur_dhakal2003@yu.ac.kr (T.D.); 2 National Institute of Ecology, Seocheon 33657, Republic of Korea; naturalist71@nie.re.kr; 3 College of Veterinary Medicine and Institute of Veterinary Science, Kangwon National University, Chuncheon 24341, Republic of Korea

**Keywords:** genomics, epidemiology, disease surveillance, whole-genome sequencing, public health

## Abstract

Genomics has revolutionized epidemiology and disease surveillance by providing powerful tools for identifying, tracking, and analyzing pathogens at the molecular level. This exploratory analysis examines the integration of genomic with traditional epidemiological approaches, highlighting the role of whole-genome sequencing as a key method for pathogen identification, outbreak investigation, and understanding transmission dynamics. By enabling the detection of mutations and monitoring of antimicrobial resistance, genomics allows for precise mapping of infection sources and transmission pathways, thereby improving the timeliness and accuracy of public health responses. Furthermore, genomic surveillance supports the early detection of emerging variants, such as those observed during viral outbreaks like COVID-19, facilitating proactive intervention strategies. Despite its transformative potential, challenges related to data privacy, infrastructure, and interdisciplinary collaboration persist. This analysis emphasizes the importance of genomics in building resilient surveillance systems to address future infectious disease threats and advocates for sustained investment in genomic technologies to advance global health security.

## 1. Introduction

Emerging infectious diseases continue to pose significant challenges to global health and biosecurity, driven by factors such as population growth, globalization, urbanization, and increased human–animal interactions [1]. These factors facilitate cross-species transmission and the emergence of novel pathogens with pandemic potential. Genomic technologies have revolutionized infectious disease surveillance by enabling the rapid identification, characterization, and monitoring of pathogens with unprecedented precision [2]. In outbreak settings, genome sequencing aids in source tracing, predicting transmission routes, and guiding targeted public health interventions. Over the past decade, genomics has become a cornerstone of outbreak investigation, surpassing traditional diagnostic tools in detecting new pathogen strains, determining their genetic composition, and predicting antimicrobial resistance (AMR) [3,4]. By analyzing pathogen genomes, researchers can identify infection sources, track transmission pathways, and design more effective control strategies [5]. Unlike conventional epidemiological approaches that rely solely on case data, genomic epidemiology integrates sequencing data with contextual metadata (e.g., time and location) to reconstruct detailed transmission networks and outbreak dynamics [6]. This integration offers more precise, evidence-based insights into disease spread and enables timely, data-driven interventions.

Advances in sequencing technologies and bioinformatics, particularly whole-genome sequencing (WGS), have greatly enhanced the capacity for high-resolution pathogen tracking and comparative genomic analysis [7,8,9]. WGS offers single-nucleotide resolution and can be applied directly to clinical or environmental samples, including blood, swabs, feces, and tissue, enabling comprehensive detection even in cases of mixed infections. The typical workflow involves nucleic acid extraction, library preparation, and sequencing on platforms such as Illumina, PacBio, or Oxford Nanopore, followed by bioinformatics pipelines for genome assembly, variant calling, and phylogenetic inference [10,11]. Complementary approaches, such as metagenomic next-generation sequencing and quantitative polymerase chain reaction (qPCR)-informed sequencing, facilitate simultaneous detection and quantification of multiple pathogens within a single sample. These methods support infection burden estimation through read-depth and coverage-based analyses [10]. Comparative genomics further distinguishes outbreak-related strains from sporadic cases, maps transmission routes, and monitors AMR determinants—especially when integrated with temporal and geographic metadata for near real-time epidemiological reconstruction.

Recent developments in portable sequencing devices, such as the Oxford Nanopore MinION, have enabled on-site genomic surveillance during field outbreaks, including those involving Ebola, COVID-19, and African swine fever. These tools generate actionable genomic data within hours, enabling rapid public health responses and containment measures [12]. Overall, WGS and related genomic technologies form the backbone of modern disease surveillance by combining high-throughput sequencing with advanced analytics to deliver precise pathogen detection, quantitative infection assessment, and critical insights into pathogen evolution and transmission dynamics. The increasing accessibility and cost-effectiveness of these technologies highlight their indispensability for early outbreak detection and control, as well as for the sustained monitoring of endemic and emerging threats.

In an era marked by emerging and re-emerging infectious diseases, the integration of genomics into epidemiology represents a pivotal advancement in global health. This study examines how genomics can be applied to enhance the detection, understanding, and control of infectious diseases through the analysis of pathogen and host genetic material. It reviews the major applications of genomics in disease surveillance, evaluates its impact on public health systems, and identifies key challenges that must be addressed to strengthen global preparedness for future infectious threats.

## 2. Materials and Methods

We conducted a scoping review of peer-reviewed studies integrating genomic data with traditional epidemiological methods for infectious disease surveillance and outbreak investigation. A Google Scholar (https://scholar.google.com/) search on 1 August 2025 using the terms “genomic epidemiology,” “whole-genome sequencing,” “genomic surveillance,” “pathogen transmission,” “outbreak investigation,” and “antimicrobial resistance” identified 220 records. After screening and full-text assessment, 30 articles met inclusion criteria, applying genomic methods such as WGS, phylogenetics, or phylodynamic and integrating findings with tools including contact tracing, case investigations, serology, or spatiotemporal analyses. Owing to heterogeneity across pathogens, settings, and analytical pipelines, we synthesized results narratively by use cases such as outbreak detection, transmission reconstruction, variant signal detection, and AMR monitoring and summarized key implementation considerations in epidemiology and pathogen surveillance.

## 3. Genomic Surveillance and Transmission Dynamics of Infectious Diseases

WGS of pathogenic genomes, combined with bioinformatics analyses, has significantly improved the speed and precision of genomic applications in outbreak tracking and infection control. Infectious diseases remain a leading cause of mortality worldwide, with viruses, bacteria, and protozoa exhibiting high mutation rates [13,14]. These rapid evolutionary changes contribute to the emergence of new human infections, more virulent strains, and antibiotic- or drug-resistant organisms. In this context, genomic surveillance serves three primary purposes: (i) to enable global monitoring of pathogens through WGS, (ii) to elucidate the development and spread of drug resistance and infectious agents, and (iii) to provide data that guide public health interventions [12,15].

Globalization and international travel complicate outbreak detection by accelerating cross-border transmission, while the co-circulation of multiple genotypes in endemic regions fosters gene flow and the emergence of variants with novel phenotypes [16]. WGS and population genomics address these challenges by resolving fine-scale spatiotemporal patterns, quantifying genetic relatedness and geographic structure, and clarifying evolutionary dynamics [17,18]. As genomic datasets expand and become increasingly integrated into surveillance systems, they continue to strengthen outbreak detection, source attribution, and response strategies [18,19].

Malaria exemplifies the value of WGS-guided surveillance and transmission analysis. Along the China–Myanmar border, *Plasmodium vivax* isolates formed a distinct local lineage, revealing ancestral uniqueness and informing geographically tailored elimination strategies [19,20,21]. Genomic analyses have also clarified parasite movement and evolution. In Malaysian Borneo, introgression between two *Plasmodium knowlesi* subpopulations associated with different macaque hosts influenced mosquito transmission potential [22]. Global *P. vivax* data indicate a South Asian origin with declining genetic diversity away from Southeast Asia, consistent with a founder effect [23]. Similarly, *P. simium* likely evolved from New World *P. vivax* through human-to-primate transmission. Moreover, machine learning applied to WGS data has identified *P. vivax* infections imported into Africa from South Asia, improving the targeting of control resources [24]. Despite sustained elimination efforts, persistent reservoirs and cross-border introductions continue to sustain transmission risk, underscoring the importance of continuous WGS monitoring for malaria control and eradication [19,20].

Arboviral genomics has likewise advanced outbreak investigations. Dengue virus (DENV-1 to DENV-4), which is endemic in more than 129 countries with major burdens in Asia, South America, and the Western Pacific [25,26], has shown increasing incidence in Africa and heightened risk in Europe and North America due to climate change and the expansion of *Aedes albopictus* [26,27]. In Florida, record dengue activity from 2022 to 2023 included both travel-related and locally acquired cases; sequencing revealed DENV-1 and DENV-3 genomes clustering with strains from India (2019), while DENV-2 aligned with a 2018 China strain, highlighting the role of international travel in sustaining transmission [28]. For the West Nile virus (WNV), Europe has experienced at least 13 independent introductions of lineages 1a and 2, with lineage 2 dominant in temperate regions such as Germany and the Netherlands; these patterns, integrated into platforms such as Nextstrain, illuminate ongoing geographic expansion and viral epidemiology [29,30,31]. A cross-pathogen summary of representative WGS applications, including regional contexts, key genomic findings, and associated public health impacts, is presented in Table 1.

Bacterial genomics has significantly improved the detection of introductions and high-risk lineages. Highly virulent methicillin-resistant *Staphylococcus aureus* (MRSA) strains, such as CC239/ST239, are widespread in Asia. In Denmark, most MRSA ST239 cases were associated with travelers returning from Asia, Africa, or the Middle East, demonstrating how WGS supports travel-related source attribution and facilitates timely infection control [33]. During hemolytic uremic syndrome epidemics, WGS of Shiga toxin-producing *Escherichia coli* has enabled real-time monitoring of emerging strains, differentiation between localized and widespread outbreaks, and mapping of transmission routes—critical steps in reducing pediatric mortality [45,46]. Genomic analysis of *E. coli* O104:H4 associated with the 2009–2011 fenugreek seed outbreak traced its origin to Egypt and revealed close genetic relatedness between French and German cases, with minor genomic variations explaining divergent epidemic trajectories [16].

Protozoan and fungal pathogens have also benefited from genomic surveillance. WGS of *Cryptosporidium hominis* subtype If A12G1R5 identified three genetically distinct, low-diversity subpopulations, suggesting the recent emergence of a hypertransmissible lineage in the United States and enabling targeted public health interventions [47]. Similarly, genomic studies of *Candida auris* in India revealed clonal isolates with low diversity across patients and hospitals. The presence of the *MATα* mating locus and transporter genes (ABC and MFS) may contribute to multidrug resistance, while its genetic distinctness from other *Candida* species indicates an independent evolutionary trajectory potentially linked to antifungal overuse [34].

Viral transmission dynamics can be elucidated through lineage-aware, region-specific analyses. For example, since its first identification in 1953, the Chikungunya virus has exhibited distinct regional patterns enzootic spillover in West Africa, recurrent introductions into East Africa from Asia (including the Indian Ocean lineage), and limited interregional viral flow all clarified by genomic studies [14,48,49]. These insights reveal hidden transmission routes and the evolutionary pressures shaping epidemic potential. Tuberculosis surveillance increasingly employs WGS to dissect transmission pathways and identify genetic determinants of fitness. Longitudinal tracking of the *Mycobacterium tuberculosis* Zaragoza (MtZ) strain, implicated in 242 cases between 2004 and 2020, identified single-nucleotide polymorphisms in genes related to virulence, pathogenesis, and survival, as well as additional mutations that may account for its unusually high transmissibility. Such findings enable more precise, strain-specific public health interventions.

Collectively, these applications illustrate how WGS and population genomics can resolve fine-scale spatiotemporal transmission patterns, uncover origins and introductions (including travel-associated events), identify resistance and virulence determinants, and distinguish localized clusters from widespread dissemination. As WGS capacity expands and global datasets grow, the integration of genomic, epidemiologic, and mobility data through phylogenomics, phylodynamics, and machine learning will remain essential for early detection of emerging threats, optimization of control strategies, and sustained progress toward disease elimination [14,49,50].

## 4. Genomics in Determinants of Drug Resistance

The widespread use of antimicrobial agents exerts strong selective pressure on pathogen populations by eliminating susceptible strains, thereby creating a population bottleneck. This process favors the survival of resistant variants and increases the frequency of drug-resistance alleles at specific loci and across linked haplotypes [14]. The emergence and spread of AMR have become one of the most pressing challenges in infectious disease control and global public health protection [51]. Advances in diagnostic microbiology have increasingly integrated conventional techniques with WGS and bioinformatics analyses, enabling the rapid identification of novel AMR genes and providing insights into their mechanisms of dissemination [52].

Recent WGS investigations have revealed numerous AMR determinants across bacterial pathogens. For example, *Neisseria gonorrhoeae* has acquired novel *mtrR* mutations and *penA* alleles conferring resistance to azithromycin and cefixime [53]. Mutations in the 23S rRNA gene of *Helicobacter pylori* have been linked to clarithromycin resistance, whereas *Proteus* species have exhibited florfenicol resistance mediated by the *floR* gene located on both plasmids and chromosomes underscoring the role of mobile genetic elements in resistance gene transfer [54,55]. Similarly, genome sequencing of multidrug-resistant *Staphylococcus lentus* identified 11 resistance genes distributed across plasmids and chromosomes [56], while tetracycline-resistant *Arthrobacter nicotianae* carried eight resistance genes, including mobile loci located outside plasmids [57].

Beyond gene discovery, WGS plays a central role in characterizing the genomic epidemiology of AMR. Analysis of 150 *N. gonorrhoeae* isolates from Ukraine revealed resistance to ciprofloxacin, tetracycline, and benzylpenicillin, with phylogenomic clustering into six major groups associated with multidrug-resistant lineages [58]. Mutations in *gyrA* S91F and *parC S87R* were correlated with ciprofloxacin resistance, while *rpsJ* V57M, *tetM*, and mosaic *penA-34.001* contributed to tetracycline and β-lactam resistance. These findings demonstrate how genomics can link specific genetic markers to resistance phenotypes, thereby improving AMR monitoring, surveillance, and outbreak management.

Genomic approaches have also deepened the understanding of drug resistance in parasitic diseases. In *Plasmodium falciparum*, the *pfcrt* K76T mutation has long served as a key marker of chloroquine resistance. Genome-wide association studies (GWAS) have identified additional variants, including *pfcrt* C350R and *pfaat1* S258L, which are associated with chloroquine resistance in French Guiana and The Gambia, respectively [59]. Beyond traditional GWAS, population genetic tools such as linkage disequilibrium mapping, site frequency spectrum analysis, and machine learning are increasingly employed to identify genetic drivers of resistance and predict emerging resistance mechanisms [60]. Open datasets for *P. falciparum* and *P. vivax* have further enabled global surveillance by cataloging resistance markers such as *pfdhfr*, *pfdhps*, *pfk13*, *pfmdr1*, *pfcrt*, and *pfama1*, thereby informing malaria control strategies [61].

In addition to bacterial and parasitic pathogens, WGS has provided valuable insights into resistance mechanisms in opportunistic organisms such as *Enterococcus faecium*. A large-scale study of 1025 bloodstream isolates from Australia identified three major genomic clusters, including both clonal complex (CC) and non-CC strains, many of which carried *vanA* and *vanB* operons conferring vancomycin resistance [62]. Distinct subclusters were associated with specific geographic regions, highlighting the value of genomic epidemiology in contextualizing resistance dynamics and predicting future trends.

Collectively, these findings highlight the transformative role of WGS in mapping the genetic determinants of drug resistance. By linking genotype to phenotype through comparative genomics, GWAS, and machine learning, genomics not only enables the discovery of novel biomarkers but also strengthens resistance surveillance systems. The integration of genomic data into public health frameworks remains essential for combating the growing threat of AMR worldwide. Representative examples of pathogens, resistance determinants, and the contributions of WGS to understanding drug resistance are summarized in Table 2.

## 5. Role of Genomics in Epidemiological Surveillance

Epidemiological surveillance involves the systematic collection, analysis, and dissemination of health data to support the planning, implementation, and evaluation of public health programs. It requires continuous monitoring of health events within populations and is typically integrated into health care systems to track major public health issues. The primary goal of surveillance is to strengthen disease monitoring and related public health activities [71]. When genomic and epidemiological data are combined, they provide near-real-time insights into outbreaks, enabling timely and targeted interventions to protect communities. Implementing genomic epidemiology across different regions and levels of health care requires coordinated collaboration to fully harness its potential for mitigating outbreaks, tracking pathogen spread, and containing emerging infectious variants.

The integration of genomic data into surveillance systems has significant practical implications, particularly for infectious disease control. WGS enables direct analysis of pathogens from clinical samples, offering critical information during outbreaks—especially regarding mutations that influence virulence, drug resistance, and antigenicity [72]. These data also enhance molecular diagnostics at the point of care and support the development of personalized treatment strategies, paralleling precision medicine approaches. At the population level, combining genomic and epidemiological data facilitates the identification of pathogen mutations associated with transmission events, providing detailed insights into infection and transmission dynamics. This approach enables the design of more precise and targeted public health interventions, surpassing the capabilities of traditional surveillance systems. Moreover, genomic epidemiology aligns with the One Health framework, which recognizes the interconnectedness of human, animal, and environmental health, thereby advancing disease surveillance, prevention, and control within a broader ecological context [4].

The World Health Organization (WHO) emphasizes the critical role of epidemiological surveillance in defining health problems, understanding established and emerging diseases, and guiding effective interventions [73]. Comprehensive surveillance systems capture essential data on pathogen clones and lineages, clinical manifestations, affected populations, and morbidity and mortality rates. Such information is fundamental for evidence-based decision-making, efficient resource allocation, and the design of effective public health interventions. Continuous surveillance is also vital for monitoring and evaluating ongoing programs [74]. Accurate systems that track the geographic spread of diseases enable assessment of health event significance and provide a foundation for informed policy and funding decisions. Genomic surveillance has proven highly effective in detecting outbreaks, tracing their origins, and monitoring AMR trends [75,76]. Long-term AMR studies highlight the value of integrating routine genomic surveillance into national and global health strategies, particularly for guiding treatment protocols and vaccination programs. Genomic data facilitate the identification of emerging AMR lineages, tracking of mobilizable resistance genes, and evaluation of genotypic resistance predictions, although limitations remain for certain pathogens [77,78]. The United Kingdom’s experience demonstrates that routine genomic sequencing of *Salmonella* has identified more outbreaks than traditional microbiological methods, illustrating the potential of genomics to enhance outbreak detection and intervention prioritization [79].

Global initiatives have increasingly standardized AMR surveillance for health care-associated infections, providing insights into dominant regional lineages and situating them within a global context [80]. Identifying emerging and prevalent AMR lineages worldwide is crucial for guiding research, informing public health responses, and optimizing control strategies. By integrating genomics into epidemiological surveillance, public health systems can detect outbreaks more rapidly, better understand transmission dynamics, and implement interventions more effectively—ultimately strengthening global health security.

## 6. Advances in Genomic Epidemiology and Pathogen Surveillance

The COVID-19 pandemic marked a pivotal turning point in global genomic surveillance. Before 2020, genomic surveillance data were rarely available to the public; however, the rapid release of *SARS-CoV-2* genomic sequences beginning on 10 January 2020, fundamentally changed this landscape. Within days, diagnostic tests were developed, with the first available by 13 January 2020 [81]. Open access to genomic epidemiological data proved critical for reconstructing transmission patterns, guiding intervention strategies, and informing control measures [82,83]. Genomic surveillance played a central role in the pandemic response by monitoring viral evolution, tracking transmission pathways, and identifying emerging variants that shaped diagnostic, therapeutic, and vaccine development.

Throughout the pandemic, the International Health Regulations (2005), Emergency Committee of the WHO emphasized the importance of strengthening genomic surveillance systems. Similarly, the WHO-convened Independent Panel for Pandemic Preparedness and Response called for sustained global investment to expand sequencing capacity. Although achieving equitable access to genomic technologies remains a challenge, maintaining progress in genomic surveillance is essential to strengthening global health security [84]. In response, the WHO launched the Global Genomic Surveillance Strategy for Pathogens with Pandemic and Epidemic Potential in March 2022, developed through extensive consultation with global, regional, and national stakeholders including public health institutions, One Health partners, and WHO technical programs the strategy outlines a 10-year roadmap for integrating genomic data into global health systems [84, 85 ].

The regional implementation of this strategy has been adapted to local needs and capacities. For instance, investments in sub regional hubs have enhanced genomic surveillance infrastructure in Europe and Africa, while in densely populated regions such as Southeast Asia, efforts focus on leveraging regional assets through consortia of policymakers, researchers, and private partners. In the Eastern Mediterranean, where political instability and humanitarian crises often impede progress, external support has been essential for establishing reference laboratories and fostering international collaboration [85]. These regionally tailored approaches allow countries to expand genomic sequencing capacity incrementally while aligning implementation with national and regional health priorities.

Technological innovations in communication have also reshaped pathogen surveillance. The widespread use of the internet, smartphones, and social media has improved both the speed and accuracy of disease detection. By integrating traditional surveillance with digital health tools such as online self-reporting systems, internet search data, and social media analytics public health authorities can identify early signals of outbreaks more rapidly [86,87,88]. These digital approaches complement genomic sequencing by providing real-time epidemiological context and broadening the reach of surveillance systems.

In parallel, WGS has advanced the prediction and characterization of AMR. Tools such as AMRFinderPlus, ResFinder, and the Comprehensive Antibiotic Resistance Database (CARD) integrate genomic data with bioinformatics to identify AMR genes, resistance-associated mutations, and gene classes [52,77]. CARD, in particular, combines curated genetic data with machine learning through CARD*Shark, which extracts information from the scientific literature. Its Resistance Gene Identifier, aligned with the Antibiotic Resistance Ontology, provides detailed insights into AMR determinants [77]. The incorporation of machine learning has enhanced the predictive accuracy and versatility of these tools across diverse pathogens and antimicrobial classes.

The rise of big data and artificial intelligence (AI) has further amplified the capabilities of genomic epidemiology. Advances in sequencing and digital health technologies have generated vast datasets that integrate genomic information, clinical records, imaging, and laboratory results. AI and machine learning algorithms are increasingly employed to uncover hidden correlations within these datasets, enabling more precise identification of biomarkers and disease associations [89]. In epidemiological surveillance, the integration of big data analytics and AI not only accelerates pathogen detection and resistance profiling but also strengthens predictive and diagnostic capacities for future outbreaks [90].

## 7. Challenges in Genomic Epidemiology

Genomic epidemiology provides substantially higher resolution than traditional surveillance methods, such as case-based reporting, exposure histories, contact tracing, serotyping, pulsed-field gel electrophoresis, multilocus sequence typing, and culture-based antimicrobial susceptibility testing. By resolving fine-scale transmission links, distinguishing co-circulating clusters, identifying introductions, and directly detecting AMR and virulence determinants, WGS enhances outbreak detection and accelerates targeted interventions compared with the discriminatory power and timelines of conventional methods [4]. Standardized data formats and analytical frameworks further promote cross-border comparability, enabling robust global situational awareness despite heterogeneity in diagnostics and case definitions. Moreover, phylogenetic and phylodynamic analyses provide critical evolutionary context, quantifying lineage turnover, recombination, and fitness changes that traditional surveillance methods cannot easily infer. These insights support vaccine and diagnostic development, as well as risk assessment [91,92,93]. However, the advantages of genomic epidemiology come with distinctive biases and operational challenges that must be carefully managed.

Compared with conventional surveillance systems, genomic inference is particularly sensitive to sampling and ascertainment biases. Sequencing efforts often over-represent severe, hospitalized, or urban cases, skewing lineage frequencies, molecular clock estimates, and transmission reconstructions. Although traditional case-based systems are also affected by care-seeking behavior and testing access, they generally maintain broader, albeit lower-resolution, coverage [94,95]. Temporal and geographic disparities in sequencing intensity can generate misleading signals of introductions or lineage extinction [96,97]. Low-pathogen-load samples (high cycle threshold [Ct] values) are frequently under sequenced, enriching datasets for acute or severe infections and biasing evolutionary and transmission inferences. Additionally, culture- or amplicon-based protocols may distort genetic diversity through in vitro adaptation or primer dropout [98,99]. Further biases arise from reference genome selection and assembly approaches. Mapping to distant reference genomes can result in the omission of novel segments or structural variants, while de novo assembly may misplace mobile genetic elements.

Recombination and within-host diversity also violate the tree-like assumptions of phylogenetic inference and, when combined with transmission bottlenecks, can obscure directionality and donor–recipient relationships that cannot be resolved from genomic data alone [100,101,102]. Additionally, model and annotation uncertainties—spanning lineage assignment, molecular clock calibration, and genotype–phenotype prediction can limit interpretability. AMR genotypes may not fully reflect phenotypic resistance due to gene expression variability or epistatic interactions. Laboratory contamination and index hopping, particularly in low-biomass samples, can generate false genomic links that are not observed in aggregated traditional surveillance data [103]. Furthermore, because genomes serve as high-resolution identifiers when paired with detailed metadata, privacy and reidentification risks surpass those typical of aggregate case reporting, necessitating stronger data governance and ethical oversight [4,104].

Mitigating these biases requires deliberate attention to study design, methodology, and interpretation. Prospective sampling frameworks that define geography, time frame, disease severity, and setting—combined with transparent reporting of denominators and minimal interoperable metadata (e.g., onset date, Ct value, travel history, care setting, and outcomes)—can stabilize phylodynamic inferences and contextualize detected clusters [103,105,106]. Wet-lab improvements, such as optimized library preparation for high-Ct samples, hybrid capture to recover low-titer genomes, updated primer schemes, replicate sequencing, and orthogonal validation assays, help reduce culture- and amplicon-derived artifacts. Analytically, the use of lineage-appropriate or graph-based references, recombination-aware phylogenetic models, haplotype-based inference, long-read confirmation where feasible, and standardized quality control pipelines with contamination safeguards can minimize reference, assembly, and crosstalk biases [105,107]. Operationally, defining explicit action thresholds such as single-nucleotide polymorphism distance and cluster growth rate criteria calibrated to substitution rates and transmission contexts—should be undertaken collaboratively with field epidemiologists. Such alignment ensures that genomic signals translate into timely and proportionate public health interventions rather than remaining confined to academic interpretation [108].

Real-world implementation also depends on infrastructure, workforce capacity, sustainability, and governance—domains in which traditional surveillance systems often face fewer barriers. Despite declining sequencing costs, many low- and middle-income countries remain underequipped, lacking sufficient laboratory facilities, sequencing platforms, bioinformatics capacity, secure data infrastructure, and trained personnel, with sustainable funding remaining limited. For example, Algeria has only one sequencing center and minimal PCR capacity, serving approximately 45 million inhabitants. During the early COVID-19 pandemic, in-country limitations at the Pasteur Institute necessitated shipping samples to the Pasteur Institute in France, delaying national data generation and public health responses. These gaps highlight the need for regional sequencing hubs, pooled procurement systems, diversified sequencing platforms, strategic buffer stocks, cloud-based analytical resources, open-source and standardized bioinformatics pipelines, and targeted training programs spanning molecular biology, bioinformatics, and epidemiology. Equally important are standard operating procedures and decision-support frameworks that integrate genomics into routine surveillance systems. Embedding genomic data into existing workflows ensures that phylogenetic findings translate into actionable public health measures, similar to how traditional incidence thresholds are institutionalized in surveillance programs [109].

Governance, data sharing, and incentive structures are also pivotal to effective implementation. Political, legal, and ethical challenges related to data ownership, attribution, privacy, intellectual property, and national security can hinder cross-border genomic data exchange essential for phylodynamic and risk assessments, even when traditional reporting occurs under the International Health Regulations. Transparent data-use agreements, privacy-preserving architectures with tiered access, attribution norms, and benefit-sharing frameworks can improve timeliness, enhance trust, and protect both individual and national interests [104,109]. Since genomic data combined with rich metadata increase the risk of reidentification, privacy-by-design principles and robust ethical oversight must accompany the scale-up of genomic surveillance systems [104].

The COVID-19 pandemic further demonstrated that human-only surveillance can overlook critical animal and environmental reservoirs, including reverse zoonotic transmission pathways that may reshape viral evolution and generate novel variants [110]. Adopting a One Health framework that integrates human, veterinary, and environmental genomics can uncover hidden transmission routes, anticipate spillover events, and strengthen pandemic preparedness by broadening sampling coverage and analytical integration across species and ecosystems [111]. Emerging computational approaches also enhance these capabilities: AI and machine learning can prioritize samples for sequencing, detect anomalous transmission patterns in real time, and predict viral evolution related to virulence, resistance, or antigenic drift. However, to be operationally effective, such models must remain transparent, well-calibrated, and continuously validated in public health contexts, with clear communication of uncertainty and alignment with decision-making frameworks [112]. Expanding and standardizing epidemiological metadata including infection source, travel history, hospitalization metrics, and outcomes will further contextualize genomic findings and refine public health priorities.

Ultimately, genomics complements rather than replaces traditional surveillance methods. When integrated with case surveillance, contact tracing, mobility data, environmental monitoring, and field investigations, WGS provides a high-resolution perspective that can validate, refine, or redirect public health interventions. Realizing its full potential requires addressing infrastructure and workforce gaps, embedding ethical safeguards and interoperable data standards, and developing joint analytic and operational playbooks to ensure that genomic insights translate into timely, equitable, and effective actions. With sustained investment, interdisciplinary coordination, and equitable access, genomic epidemiology can fulfill its promise of transforming disease surveillance and response across both routine and emergency contexts [104,109,112].

## 8. Conclusions

The integration of genomics into public health surveillance has fundamentally transformed the management of infectious diseases, providing unprecedented precision in detecting, tracking, and responding to pathogens. This review highlights the pivotal role of WGS and other genomic technologies in advancing infectious disease surveillance and epidemiology. Evidence consistently demonstrates that genomics delivers high-resolution insights into pathogen transmission, evolution, and AMR, thereby enhancing the accuracy and effectiveness of public health interventions. However, realizing the full potential of these technologies requires addressing persistent challenges related to infrastructure, data governance, workforce capacity, and equitable access. Continued investment, interdisciplinary collaboration, and global coordination are essential to harness genomics for building resilient, data-driven public health systems capable of responding effectively to both current and emerging infectious disease threats. By enabling more timely, accurate, and targeted interventions, genomics has become an indispensable component of modern outbreak response strategies. Sustained efforts to strengthen infrastructure, promote equitable access, and foster cross-disciplinary collaboration will be critical for maximizing its long-term impact. This study serves as a resource for scholars, researchers, and policymakers seeking to understand how genomics can shape effective, data-driven infectious disease surveillance systems and inform strategies for global health preparedness.

## Figures and Tables

**Table 1:** Applications of Whole-Genome Sequencing (WGS) in Disease Surveillance and Outbreak Investigation.

Pathogen	Region/Context	Key WGS Findings	Public Health Impact	Reference
*Plasmodium vivax*	China–Myanmar border, Greater Mekong Subregion	Distinct local genetic clade identified; clustering confirmed by ML and IBD analyses	Informs geographically targeted malaria elimination strategies; highlights risk of parasite resurgence	[20]
Dengue virus (DENV-1–4)	Nepal; Asia, Africa, Americas, Europe	Genomes linked to strains from India (2019) and China (2018); global movement detected	Traces outbreak sources; supports control strategies in endemic and newly affected regions	[28]
West Nile virus (WNV)	Europe and North America	≥13 introductions in Europe; lineage 2 expanding into temperate regions	Improves understanding of viral evolution and spread; informs surveillance priorities	[32]
Methicillin-resistant *Staphylococcus aureus* (MRSA)	Asia, Africa, Middle East, Europe	Travel-associated transmission detected; genomic tools outperform traditional typing	Supports infection control policies and strengthens cross-border AMR surveillance	[33]
*Candida auris*	India	Clonal strains with low genetic diversity; multidrug resistance linked to transporter genes	Identifies antifungal resistance mechanisms; guides hospital infection control measures	[34]
*Escherichia coli* ST131	Global (e.g., Europe, Asia, North America)	Dominant clone associated with fluoroquinolone resistance; rapid global spread	Informs AMR surveillance and control strategies	[35]
*Vibrio parahaemolyticus*	Asia; Gulf of Mexico	Identified oceanic gene pools; frequent recombination and host adaptation	Enhances understanding of environmental reservoirs and transmission dynamics	[36]
*Salmonella enterica*	United States	Emergence of epidemic strains; network-based genomic analysis	Improves outbreak detection and source attribution in foodborne illnesses	[37]
*Campylobacter jejuni*	Australia, Europe, North America	Evidence of rapid host switching; challenges in source attribution	Guides public health interventions and food safety measures	[38]
*Clostridium perfringens*	Global	High genetic diversity; identification of enterotoxin-producing strains	Assists in food safety and public health responses to foodborne outbreaks	[39]
Influenza virus	Global	Dynamic genome evolution; identification of novel circulating strains	Supports vaccine development and pandemic preparedness strategies	[40]
*Listeria monocytogenes*	United States	Identification of outbreak sources; improved traceback capability	Strengthens food safety measures and outbreak response strategies	[41]
Zika virus	Americas, Southeast Asia	Traced spread via mosquito vectors; identified mutations linked to microcephaly	Informs vector control strategies and public health responses	[42]
Norovirus	India (Pune)	Mutated GII.16[P16] strain linked to Guillain–Barré syndrome outbreak	Highlights need for enhanced surveillance of neurological complications	[43]
Foot-and-mouth disease virus (FMDV)	Asia	Revealed within-host viral diversity; identified mutations linked to tissue adaptation	Improves understanding of viral evolution and transmission dynamics	[44]

**Table 2:** Selected Case Studies of WGS in Identifying Antimicrobial Resistance Determinants.

Pathogen	Key Resistance Genes/Mutations	Drug Resistance	WGS Contribution	Reference
*Neisseria gonorrhoeae*	*mtrR* mutations, *penA* alleles	Azithromycin, cefixime	Identified novel mutations associated with treatment failure	[53]
*Helicobacter pylori*	23S rRNA mutations	Clarithromycin	Linked specific mutations to macrolide resistance	[54]
*Proteus* spp.	*floR* (plasmid + chromosome)	Florfenicol	Highlighted role of mobile genetic elements in gene transfer	[55]
*Staphylococcus lentus*	11 resistance genes (plasmid + chromosome)	Multidrug resistance	Defined genomic basis of MDR	[56]
*Arthrobacter nicotianae*	8 resistance genes, mobile loci outside plasmids	Tetracycline	Identified extrachromosomal AMR genes	[57]
*Neisseria gonorrhoeae* (Ukraine isolates)	GyrA S91F, ParC S87R, *rpsJ* V57M, *tetM*, *penA*-34.001	Ciprofloxacin, tetracycline, β-lactams	Phylogenomic analysis revealed MDR lineages and key mutations	[58]
*Plasmodium falciparum*	*pfcrt* K76T, C350R, *pfaat1* S258L, *pfdhfr*, *pfdhps*, *pfk13*, *pfmdr1*, *pfama1*	Chloroquine, artemisinin, multidrug resistance	GWAS + WGS identified novel molecular markers for surveillance	[60]
*Enterococcus faecium*	*vanA*, *vanB* operons	Vancomycin	WGS of 1025 isolates defined clonal clusters, geographic subclusters, and resistance determinants	[62]
*Acinetobacter baumannii*	OXA-23, OXA-24, OXA-58 carbapenemases	Carbapenems	Characterized global spread and clonal expansion of resistant strains	[63]
*Klebsiella pneumoniae*	KPC, NDM, OXA-48 carbapenemases	Carbapenems	Identified hypervirulent strains with multidrug resistance	[64]
*Mycobacterium tuberculosis*	*rpoB* mutations, *katG* mutations	Rifampicin, isoniazid	Whole-genome sequencing for rapid detection of resistance	[65]
*Salmonella enterica*	*bla*CTX-M, *bla*TEM, *bla*SHV	Extended-spectrum cephalosporins	Tracked spread of resistance genes in foodborne outbreaks	[66]
*Shigella spp.*	*bla*CTX-M, *aac*(3)-IV	Aminoglycosides, cephalosporins	Identified plasmid-mediated resistance mechanisms	[67]
*Pseudomonas aeruginosa*	VIM, IMP, NDM metallo-β-lactamases	Carbapenems	Characterized resistance profiles and clonal spread	[68]
*Streptococcus pneumoniae*	*ermB*, *mefA*	Macrolides	Identified genetic determinants of macrolide resistance	[69]
*Enterobacteriaceae*	*bla*NDM, *bla*KPC, *bla*OXA-48	Carbapenems	Whole-genome sequencing for surveillance of resistant strains	[70]

## Data Availability

The original contributions presented in this study are included in the article. Further inquiries can be directed to the corresponding authors.
